# Silicon dioxide nanoparticles induce insulin resistance through endoplasmic reticulum stress and generation of reactive oxygen species

**DOI:** 10.1186/s12989-019-0327-z

**Published:** 2019-11-07

**Authors:** Hailong Hu, Xingpei Fan, Qian Guo, Xiangjuan Wei, Daqian Yang, Boya Zhang, Jing Liu, Qiong Wu, Yuri Oh, Yujie Feng, Kun Chen, Liping Hou, Ning Gu

**Affiliations:** 1 School of Life Science and Technology, State Key Laboratory of Urban Water Resource and Environment, Harbin Institute of Technology, No. 92 West Da-zhi Street, Harbin, Heilongjiang 150001 China; 2 Faculty of Education, Wakayama University, Wakayama, Japan; 3 State Key Laboratory of Urban Water Resource and Environment, Harbin Institute of Technology, Harbin, China; 4 The Joint Research Center of Guangzhou University and Keele University for Gene Interference and Application, School of Life Science, Guangzhou University, Guangzhou, China; 5 School of Life Sciences, Guangzhou University, Guangzhou, China

**Keywords:** Silicon dioxide nanoparticles, RNA-sequencing, Endoplasmic reticulum stress, Blood glucose, Mice

## Abstract

**Background:**

Silicon dioxide nanoparticles (SiO_2_ NPs) are one of the most widely utilized NPs in various food sectors. However, the potential endocrine toxicity of SiO_2_ NPs has not been characterized.

**Results:**

In the present study, mice were orally administered a series of doses of SiO_2_ NPs. All doses of SiO_2_ NPs were absorbed into the blood, liver, and pancreas of the mice. Administration of 100 mg/kg bw (body weight) of SiO_2_ NPs significantly increased blood glucose levels in mice. However, the same dose of SiO_2_ fine-particles (FPs) did not result in altered blood glucose. Whole-genome analysis showed that SiO_2_ NPs affected the expression of genes associated with reactive oxygen species (ROS) production and endoplasmic reticulum (ER) stress. In addition, we showed that SiO_2_ NPs activated xenobiotic metabolism, resulting in ER stress. Endoplasmic reticulum stress resulted in increased ROS production, which activated the NF-κB pathway leading to expression of inflammatory cytokines. Increased inflammatory cytokine expression resulted in serine phosphorylation of IRS1, which induced insulin resistance (IR). Furthermore these inflammatory cytokines activated the MAPK pathway, which further promoted the serine phosphorylation of IRS1. Insulin resistance resulted in elevated blood glucose. The ER stress inhibitor 4-phenylbutyric acid (4-PBA) inhibited SiO_2_ NP-induced ROS production. The ROS scavenger N-acetylcysteine (NAC) did not affect SiO_2_ NP-induced ER stress, but inhibited SiO_2_ NP-induced activation of the NF-κB and MAPK pathways, expression of inflammatory cytokines, SiO_2_ NP-induced serine phosphorylation of IRS1, and SiO2 NP-induced elevations of blood glucose.

**Conclusion:**

Silicon dioxide NPs induced IR through ER stress and generation of ROS, but SiO_2_ FPs did not. Therefore, lifelong exposure of humans to SiO_2_ NPs may result in detrimental effects on blood glucose. The results of this study strongly suggested that non-nanoformed SiO_2_ should be used as food additives.

## Background

Particles between 1 and 100 nm in size are characterized as nanoparticles (NPs) [1]. Amorphous silicon dioxide (SiO_2_) NPs have been widely used for various applications, such as coatings, paints, adhesives, composites, cosmetics, food additives, and for drug delivery and diagnostics. The conventional form of amorphous silica is used as a food additive [1, 2]. Studies have estimated the typical doses intake of SiO_2_ NPs at 124 mg per day based on consumption of food products that contain SiO_2_ NPs. The major sources of SiO_2_ NPs are noodles, soups, rubs, and coffee creamers [3, 4]. However, the potential toxicity of SiO_2_ NPs has not been addressed. In cells, exposure to SiO_2_ NPs resulted in glutathione depletion and DNA damage, and activation of the MAPK/ERK1/2 and Nrf2/ARE pathways [5, 6]. Inductively coupled plasma-mass spectrometry (ICP-MS) analysis showed that rats exposed to 2500 mg/kg body weight (bw) SiO_2_ NPs per day for 84 days by oral administration had SiO_2_ NPs distributed in the lungs, kidneys, and spleen. Although no silicon was detected in the liver, histopathological analysis and gene expression studies showed evidence of liver fibrosis after 84 days of exposure [7]. In BALB/c mice exposed to 50 nm SiO_2_ NPs, histopathological analysis showed lung thrombosis, cardiac wall fibrosis and calcifications, brain infarctions with necrotizing inflammatory response, retinal injuries with calcification, and focal gliosis [8]. In addition, blood biochemical parameters such as albumin, cholesterol, triglycerides, total protein, urea, high-density lipoprotein (HDL), and low-density lipoprotein (LDL), and alkaline phosphatase (ALP) and aspartate aminotransferase (AST) activities, were significantly increased in mice administered SiO_2_ NPs [9]. These studies showed that SiO_2_ NPs can induce wide-ranging systemic effects. However, the effects of SiO_2_ NPs on the endocrine system are unknown. Diabetes is a multifactorial endocrine disease in which both genetic predisposition and environmental factors contribute to disease onset and progression [10]. Diabetes is the fifth leading cause of death in the United States, and the number of people with diabetes in the world is expected to double from 2000 to 2030 [11]. Food additives are considered important pathogenic factors for diabetes [10, 12]. For example, the relationship between iron and glucose metabolism has been demonstrated. Excess iron can induce insulin resistance (IR) and diabetes through increased oxidative stress [12]. Excessive reactive oxygen species (ROS) production is a hallmark of oxidative damage in many diseases, and can lead to mitochondrial dysfunction, cellular aging, and apoptosis [13]. Reactive oxygen species can contribute to development of diabetes through induction of apoptosis in the pancreas and insulin resistance (IR) in the liver. Previous studies have demonstrated that SiO_2_ NPs induced cytotoxicity through excessive ROS production [6, 8]. Therefore, further investigation of the role of SiO_2_ NPs in food additives in the development and progression of diabetes is of importance. The mechanisms by which SiO_2_ NPs increase ROS levels in animals have not been characterized. Recent studies showed that ROS generation is closely linked to endoplasmic reticulum (ER) stress [14, 15]. ER stress is defined as an imbalance between the protein folding capacity of the ER, and the functional demand that is placed on this organelle. To restore ER homeostasis, cells trigger the ER stress response, also known as the unfolded protein response (UPR) [15, 16]. ER stress can either activate or deactivate the expression of vital genes associated with ROS generation, such as those in the mitochondrial respiratory chain, arachidonic acid metabolism pathway, cytochrome P450 (CYP) family, glucose oxidase, amino acid oxidases, xanthine oxidase, NADPH/NADPH oxidases, and NO synthases [16, 17]. In addition, the transcriptional factor nuclear factor E2-related factor 2 (Nrf2), a downstream effector of ER stress, is an important endogenous antioxidant pathway that protects against oxidative stress [15, 18]. However, whether ER stress is involved in ROS production in response to SiO_2_ NPs has not been reported. In this study, we evaluated the effects of dietary SiO_2_ NPs on blood glucose homeostasis. Silicon dioxide NPs were administered orally to mice, and blood glucose was measured. RNA sequencing was used to evaluate the whole-transcriptome response of the mice, and the genome-wide pathways affected by SiO_2_ NPs in mice were further investigated, particularly those related to blood glucose homeostasis.

## Results

### Characterization and absorption of SiO_2_ NPs

**Fig. 1:**
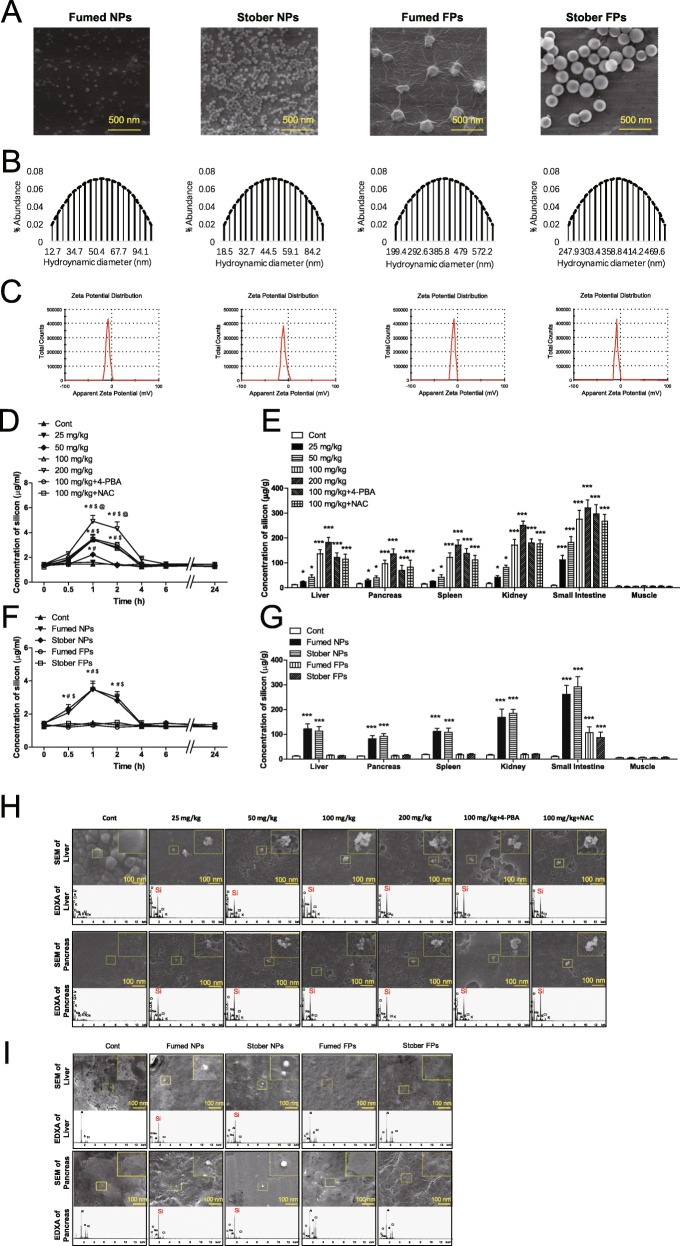
Characterization of fumed SiO_2_ NPs (nanoparticles), stober SiO_2_ NPs, fumed SiO_2_ FPs (fine-particles), and stober SiO_2_ FPs in the orally administered dose, silicon concentrations in mouse tissues, and absorption of SiO_2_ NPs in mouse tissues. **a** SEM of fumed SiO_2_ NPs, stober SiO_2_ NPs, SiO_2_ FPs, and stober SiO_2_ FPs. **b** Hydrodynamic size of SiO_2_ NPs, stober SiO_2_ NPs, SiO_2_ FPs, and stober SiO_2_ FPs. **c** Zeta potential of SiO_2_ NPs, stober SiO_2_ NPs, SiO_2_ FPs, and stober SiO_2_ FPs. **d** Silicon concentrations in blood after oral administration of different dose of fumed SiO_2_ NPs to mice. * *P* < 0.05 vs. the control group. # *P* < 0.05 vs. the 25 mg/kg group. $ *P* < 0.05 vs. the 50 mg/kg group. @ *P* < 0.05 vs. the 100 mg/kg group (*n* = 5). **e** Silicon concentrations in tissues after oral administration of different dose of fumed SiO_2_ NPs to mice for 18 weeks. * *P* < 0.05, *** *P* < 0.001 vs. the control group. Results are the mean ± SE (*n* = 10). **f** Silicon concentrations in blood after oral administration of fumed SiO_2_ NPs, stober SiO_2_ NPs, SiO_2_ FPs, and stober SiO_2_ FPs to mice. * *P* < 0.05 vs. the control group. # *P* < 0.05 vs. the Fumed FPs group. $ *P* < 0.05 vs. the stober FPs group (*n* = 5). **g** Silicon concentrations in tissues after oral administration of fumed SiO_2_ NPs, stober SiO_2_ NPs, SiO_2_ FPs, and stober SiO_2_ FPs to mice for 18 weeks. * *P* < 0.05, *** *P* < 0.001 vs. the control group. Results are the mean ± SE (*n* = 10). **h** SEM (nanosized spherical white objects are SiO_2_ NPs) and EDXA (arrows indicate silicon) of tissue homogenates after oral administration of different dose of fumed SiO_2_ NPs to mice for 18 weeks. **i** SEM (spherical white objects are SiO_2_ NPs or FPs) and EDXA (arrows indicate silicon) of tissue homogenates after oral administration of different dose of fumed SiO_2_ NPs, stober SiO_2_ NPs, SiO_2_ FPs, and stober SiO_2_ FPs to mice for 18 weeks

In this study, fumed SiO_2_ NPs (Fumed NPs) were obtained from Sigma Co. Ltd. The structure was amorphous (as indicated by the absence of peaks in the X-ray diffraction pattern), and 3.5–4.5 hydroxyl groups were present per square millimicron of SiO_2_ surface. As controls, stober SiO_2_ NPs (Stober NPs), fumed SiO_2_ fine-particles (Fumed FPs) and stober SiO_2_ fine-particles (Stober FPs) were also used in this study. Scanning electron microscopy (SEM), transmission electron microscopy (TEM), and dynamic light scattering (DLS) were used to characterize SiO_2_ NPs and FPs. The SEM and TEM images showed near-spherical shape and good dispersion of the SiO_2_ NPs and FPs. The average diameters of the Fumed NPs, Stober NPs, Fumed FPs, and Stober FPs were 35.76 ± 4.71 nm, 31.65 ± 2.33 nm, 227.63 ± 9.85 nm, and 213.37 ± 3.04 nm, respectively (Fig. 1a). The hydrodynamic sizes of the Fumed NPs, Stober NPs, Fumed FPs, and Stober FPs in PBS were 56.89 ± 4.81 nm, 51.16 ± 4.47 nm, 409.11 ± 10.37 nm, and 386.50 ± 7.99 nm, respectively (Fig. 1b). All zeta potentials were negative (Fig. 1c). The SEM and TEM results suggested that SiO_2_ NPs were well-dispersed. The level of endotoxin in all SiO_2_ NPs and FPs was below 0.01 EU/ml for all oral dose, these levels of contaminating endotoxin were determined to be inconsequential for these studies. To analyze absorption of SiO_2_ NPs at different concentrations, mice were orally administered Fumed NPs (0, 25, 50, 100, or 200 mg/kg bw). Mice were also treated simultaneously with 4-PBA (4-phenylbutyric acid) and NAC (N-acetyl-cysteine) in addition to 100 mg/kg bw Fumed NPs. Blood was collected at 0, 0.5, 1, 2, 4, 6, 8, 10, 12, and 24 h. Blood silicon levels increased and peaked at 1 h after oral administration (Fig. 1d). Then Fumed NPs were orally administered daily to mice. At the end of week 18, silicon levels were significantly increased in the livers, pancreases, spleens, kidneys, and small intestines in the groups treated with 25, 50, 100, and 200 mg/kg bw Fumed NPs compared to those in control mice (Fig. 1e). In addition, 4-PBA and NAC did not affect the absorption of SiO_2_ NPs in mice (Fig. 1d, e). To compare the absorption of different types and sizes of silicon NPs, mice were orally administrated Fumed NPs, Stober NPs, Fumed FPs, and Stober FPs (100 mg/kg bw each). Blood was collected at 0, 0.5, 1, 2, 4, 6, 8, 10, 12, and 24 h. Silicon levels increased, then peaked at 1 h after oral administration of Stober NPs, but did not change following oral administration of Fumed FPs and Stober FPs (Fig. 1f). Then, Fumed NPs, Stober NPs, Fumed FPs, and Stober FPs were orally administered daily to mice. At the end of week 18, silicon levels were significantly increased in the livers, pancreases, spleens, kidneys, and small intestines in the groups treated with 100 mg/kg of Fumed NPs and Stober NPs, and silicon levels were not significantly different between these two groups (Fig. 1g). However, silicon levels in the livers, pancreases, spleens, and kidneys of the Fumed FPs and Stober FPs groups were similar to those in the control group (Fig. 1 g). Scanning electron microscopy (SEM) and energy dispersive X-ray analysis (EDXA) showed that SiO_2_ NPs (nanosized spherical white objects), and not larger aggregates, were found in the livers, pancreases, spleens, kidneys, and small intestines of mice treated with 25, 50, 100, and 200 mg/kg bw Fumed NPs (Fig. 1h and Additional file 1 Figure S1a). In addition, scanning electron microscopy and EDXA showed that SiO_2_ NPs (nanosized spherical white objects), and not larger aggregates were found in the livers, pancreases, spleens, kidneys, and small intestines in the Fumed NPs and Stober NPs groups, but not in the livers, pancreases, spleens, and kidneys in the Fumed FPs or Stober FPs groups (Fig. 1i and Additional file 1 Figure S1b). The silicon levels in the small intestines in the Fumed FPs and Stober FPs groups were significantly increased, and SEM and EDXA showed that SiO_2_ FPs (fine-sized spherical white objects) were found in the small intestines in both groups. This observation may have been due to residual SiO_2_ FPs in the small intestines (Fig. 1g and Additional file 1 Figure S1b).

### Effects of SiO_2_ NPs on blood glucose

**Fig. 2:**
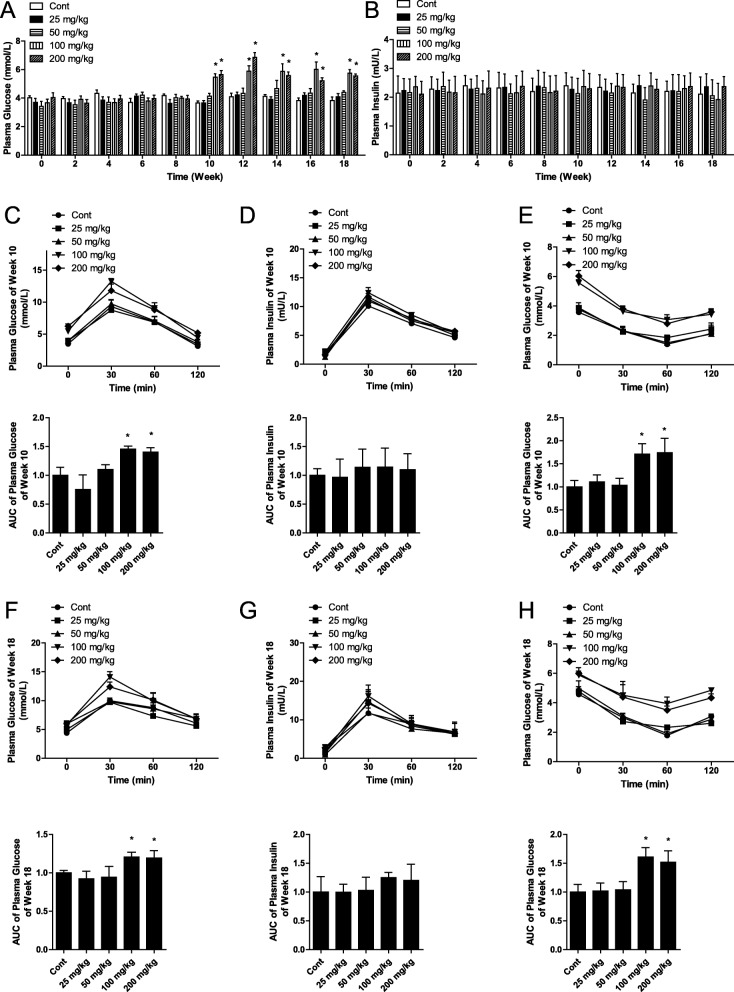
Oral administration of 100 mg/kg bw SiO_2_ NPs (nanoparticles) or greater resulted in elevated blood glucose via induction of IR (insulin resistance) in mice. **a** Blood glucose levels in blood from mice tail veins after mice orally administrated with different dose of fumed SiO_2_ NPs. **b** Blood insulin levels in blood from mice tail veins after mice orally administrated with fumed SiO_2_ NPs, stober SiO_2_ NPs, fumed SiO_2_ FPs, stober SiO_2_ FPs. **c** Time course of changes, including the AUC (area under the curve), in blood glucose levels during the OGTT at week 10. **d** Time course of changes, including the AUC, in blood insulin levels during the OGTT at week 10. **e** Time course of changes, including the AUC, in blood glucose levels during the ITT at week 10. **f** Time course of changes, including the AUC, in blood glucose levels during the OGTT at week 18. **g** Time course of changes, including the AUC, in blood insulin levels during the OGTT at week 18. **h** Time course of changes, including the AUC, in blood glucose levels during the ITT at week 18. * *P* < 0.05 vs. the control group. Results are the mean ± SE (*n* = 10)

**Fig. 3:**
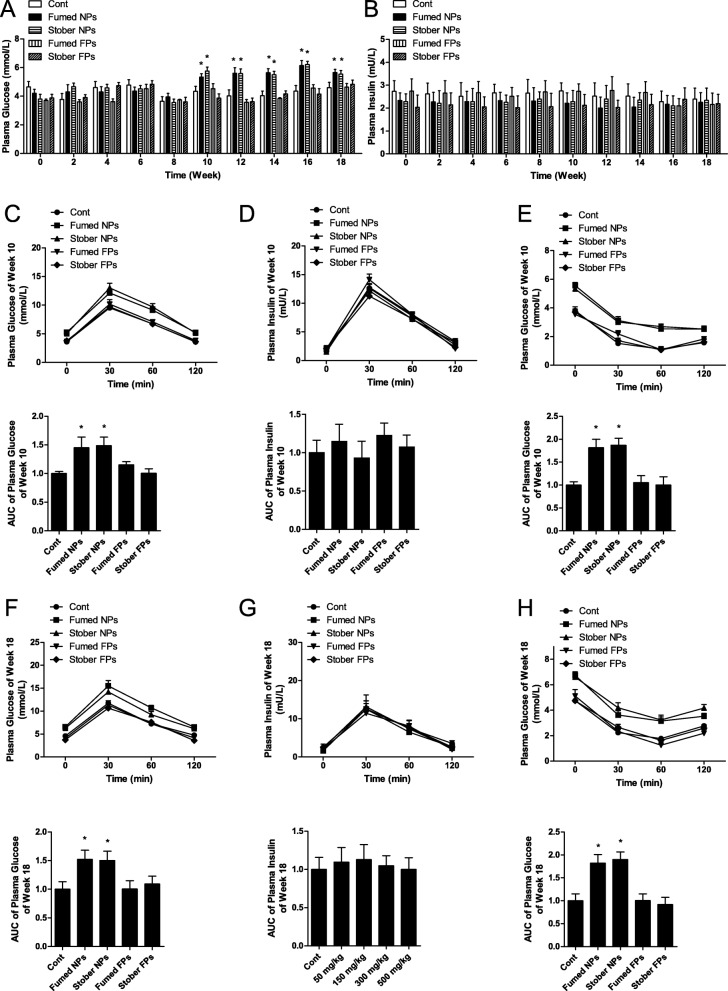
Oral administration of 100 mg/kg bw SiO_2_ FPs (fine-particles) did not result in elevated blood glucose via induction of IR (insulin resistance) in mice. **a** Blood glucose levels in blood from mice tail veins after mice orally administrated with fumed SiO_2_ NPs, stober SiO_2_ NPs, fumed SiO_2_ FPs, stober SiO_2_ FPs. **b** Blood insulin levels in blood from mice tail veins after mice orally administrated with fumed SiO_2_ NPs, stober SiO_2_ NPs, fumed SiO_2_ FPs, stober SiO_2_ FPs. **c** Time course of changes, including the AUC (area under the curve), in blood glucose levels during the OGTT at week 10. **d** Time course of changes, including the AUC, in blood insulin levels during the OGTT at week 10. **e** Time course of changes, including the AUC, in blood glucose levels during the ITT at week 10. **f** Time course of changes, including the AUC, in blood glucose levels during the OGTT at week 18. **g** Time course of changes, including the AUC, in blood insulin levels during the OGTT at week 18. **h** Time course of changes, including the AUC, in blood glucose levels during the ITT at week 18. * *P* < 0.05 vs. the control group. Results are the mean ± SE (*n* = 10)

Blood was collected from the tail veins of mice during the oral administration phase to measure blood glucose. At doses of 100 mg/kg bw and higher of Fumed NPs, blood glucose increased significantly starting at week 10. However, administration of 25 and 50 mg/kg bw of SiO_2_ NPs did not affect blood glucose (Fig. 2a). Insulin secretion was similar in each group in this study (Fig. 2b). Administration of 100 mg/kg bw of Stober NPs also increased blood glucose in mice starting at week 10, but did not affect insulin secretion (Fig. 3a and b). Administration of 100 mg/kg bw Fumed FPs or Stober FPs did not affect blood glucose or insulin secretion (Fig. 3a and b). The oral glucose tolerance test (OGTT) conducted at weeks 10 and 18 showed that the areas under the curves (AUC) resulting from administration of 100 and 200 mg/kg bw of Fumed NPs were significantly higher than those in response to administration of 25 and 50 mg/kg bw of Fumed NPs, which indicated that administration of 100 and 200 mg/kg bw of Fumed NPs resulted in reduced glucose tolerance (Fig. 2c, f). Insulin levels were similar in all groups (Fig. 2d, g). In addition, the insulin tolerance test (ITT), conducted at weeks 10 and 18, also showed that administration of 100 and 200 mg/kg bw of Fumed NPs resulted in reduced insulin sensitivity (Fig. 2e, h). The OGTT and ITT conducted at weeks 10 and 18 also showed that administration of 100 mg/kg of Stober NPs induced IR in mice, but Fumed FPs and Stober FPs did not (Fig. 3c-h). Terminal deoxynucleotidyl transferase-mediated dUDP nick end-labeling (TUNEL) results showed no apoptotic cells in the pancreases of the mice in any group at either 10 or 18 weeks (Fig. S2a). These results showed that mice that received 100 and 200 mg/kg bw of Fumed NPs exhibited insulin resistance (IR), resulting in increased blood glucose starting at week 10.

### RNA sequencing and transcriptome characterization of mice following oral administration of SiO_2_ NPs

**Fig. 4:**
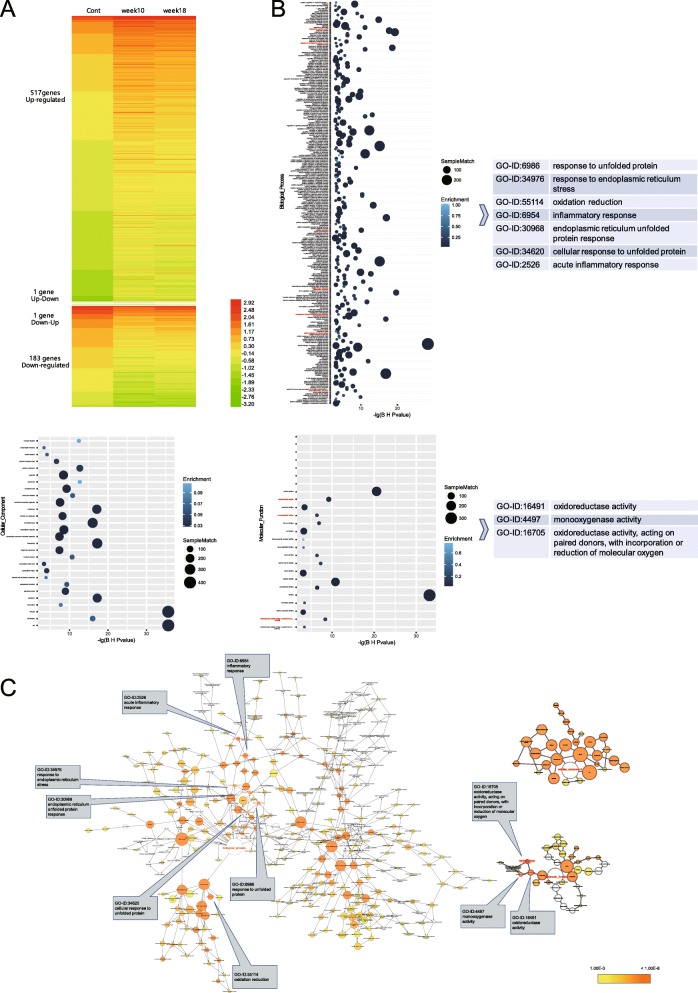
RNA-sequencing results show that oral administration of 100 mg/kg bw of SiO_2_ NPs (nanoparticles) significantly upregulated the expression of 517 genes and downregulated the expression of 183 genes at weeks 10 and 18. These genes were enriched in 311 GO (Gene Ontology) terms, including 258 BP (Biological Process) terms, 28 CC (Cellular Component) terms, and 25 MF (Molecular Function) terms. **a** Genes with significant changes in expression. **b** GO terms (BP, CC, and MF from left to right). **c** Relational network of all GO terms (BP, CC, and MF from left to right). (*n* = 3)

The livers of three mice exposed to 100 mg/kg bw of Fumed NPs for 10 and 18 weeks were analyzed using RNA-seq. The reading frames were mapped to the mouse genome (version NCBIM37/mm9). The transcriptomic analyses showed significant changes in mRNA abundance for 702 genes following oral administration of Fumed NPs, with 517 genes upregulated and 183 genes downregulated. In addition, one gene was upregulated after mice were exposed to Fumed NPs for 10 weeks, but then downregulated after 18 weeks of exposure. In contrast, one gene was downregulated after 10 weeks of exposure to SiO_2_ NPs, but then upregulated after 18 weeks of exposure (Fig. 4a and Additional file 2 Table S1). Enrichment analysis was conducted on the 517 upregulated genes and the 183 downregulated genes. The resulting agglomerative hierarchical clustering diagram contained 311 Gene Ontology (GO) terms, including 258 Biological Process (BP) terms, 28 Cellular Component (CC) terms, and 25 Molecular Function (MF) terms. These GO terms showed that SiO_2_ NPs affected the generation of reactive oxygen species (ROS) and other oxidants (GO-ID: 0055114, 16,491, 16,705, 4497, etc.), endoplasmic reticulum (ER) stress (GO-ID: 34976, 6986, 34,620, 30,968, etc.), and the inflammatory response (GO-ID: 0006954, 2526, etc.) (Fig. 4b, c and Additional file 3 Table S2).

### SiO_2_ NPs increased blood glucose in mice from week 10

**Fig. 5:**
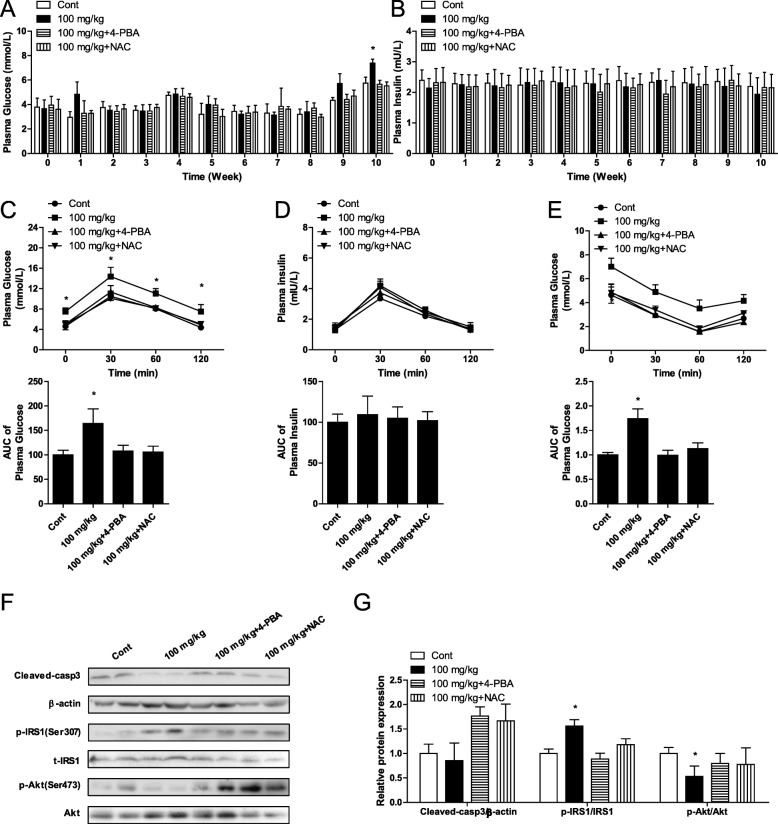
Oral administration of 100 mg/kg of SiO_2_ NPs (nanoparticles) increased blood glucose starting at week 10, and inhibition of ER stress and reduced ROS production inhibited increased blood glucose levels. **a** Blood glucose. **b** Blood insulin. **c** Time course of changes, including the AUC, in blood glucose levels during the OGTT at week 10. **d** Time course of changes, including the AUC, in blood insulin levels during the OGTT at week 10. **e** Time course of changes, including the AUC, in blood glucose levels during the ITT at week 10. **f** Protein expression of cleaved caspase 3, and phosphorylation of IRS1 and Akt. **g** Ratios of cleaved caspase 3/β-actin, p-IRS1/IRS1, and p-Akt/Akt. * *P* < 0.05 vs. the control group. Results are the mean ± SE (*n* = 10)

To analyze the relationship between the SiO_2_ NP-induced increase in blood glucose and ROS and ER stress, mice were orally administered SiO_2_ NPs, 4-PBA, and NAC. The results showed that blood glucose increased from week 10 in the 100 mg/kg bw SiO_2_ NP group (Fig. 5a). Insulin secretion was similar in each group (Fig. 5b). Glucose tolerance was tested at week 10, and SiO_2_ NPs did not affect insulin secretion in mice. However, SiO_2_ NPs impaired glucose tolerance, which was rescued by 4-PBA and NAC (Fig. 5c, d). Similarly, SiO_2_ NPs reduced insulin sensitivity in mice at week 10, as evidenced by the ITT (Fig. 5e). TUNEL results showed that SiO_2_ NPs induced cellular apoptosis, but did not affect the protein expression of cleaved-caspase 3 in liver cells of mice (Additional file 1 Figure S2b), but increased phosphorylation levels of IRS1 and reduced phosphorylation levels of Akt in liver cells (Fig. 5f, g). 4-phenylbutyric acid inhibits ER stress and NAC is a ROS scavenger. In this study, both 4-PBA and NAC effectively inhibited the effects of SiO_2_ NPs on blood glucose, glucose tolerance, insulin sensitivity, and phosphorylation of IRS1 and Akt (Fig. 5a-g). In addition, Stober NPs did not affect the protein expression of cleaved caspase 3, but altered the phosphorylation of IRS1 and Akt in liver cells of mice after 18 weeks of exposure (Additional file 1 Figure S2c, d). However, neither Fumed FPs nor Stober FPs affected the protein expression of cleaved caspase 3 or phosphorylation levels of IRS1 and Akt in mice.

### SiO_2_ NPs increased ROS levels in mice starting at week eight

**Fig. 6:**
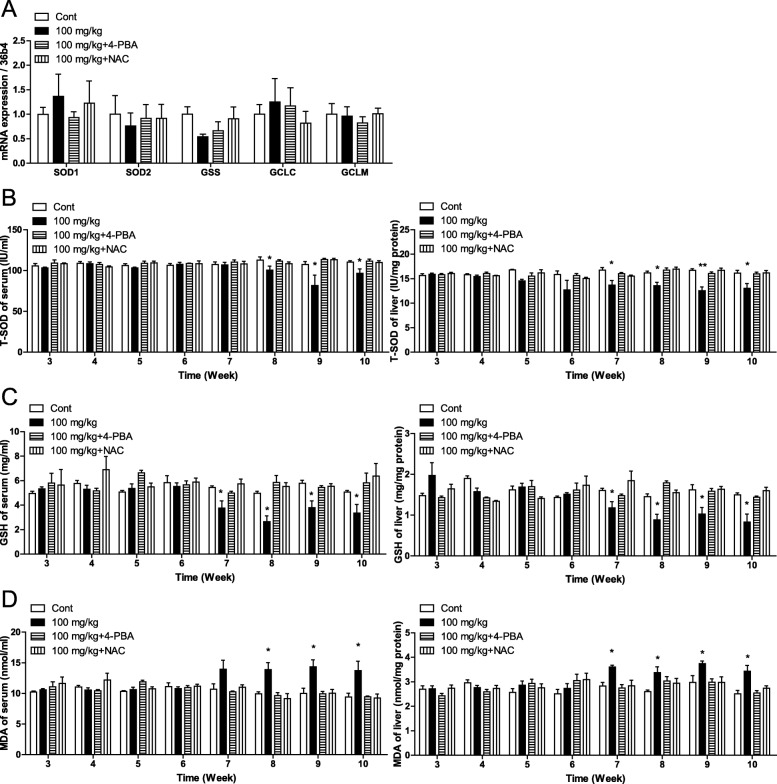
Oral administration of 100 mg/kg of SiO_2_ NPs (nanoparticles) increased plasma ROS levels starting at week eight, and inhibition of ER stress prevented increased ROS generation. **a** RT-qPCR results for ROS-related genes. **b** Levels of T-SOD in sera and livers. **c** Levels of GSH in sera and livers. **d** Levels of MDA in sera and livers. * *P* < 0.05 vs. the control group. Results are the mean ± SE (*n* = 10)

RNA-seq results showed that SiO_2_ NPs affected the generation of ROS in mice (Fig. 4b, c). RT-qPCR results showed that SiO_2_ NPs did not affect the mRNA expression of SOD1, SOD2, GSS, GCLC, or GCLM, which are genes that encode superoxide dismutase (SOD) and glutathione (GSH) (Fig. 6a). However, the levels of SOD and GSH were significantly reduced in sera and livers of mice starting at week eight (Fig. 6b, c). Furthermore levels of malonyl dialdehyde (MDA), a product of lipid peroxidation, were significantly increased in sera and livers of mice starting at week eight (Fig. 6d). These results suggested that SiO_2_ NPs increased ROS levels in mice starting at week eight. In addition, both 4-PBA and NAC effectively inhibited the effects of SiO_2_ NPs on ROS levels in sera and livers of mice (Fig. 6a-d). Nqo1, Nrf2, and HO-1 are genes in the Nrf2 pathways associated with ROS generation. In this study, RNA-seq results showed that SiO_2_ NPs affected the mRNA expression of Nqo1, Nrf2, and HO-1 (Fig. 6e). The effects of SiO_2_ NPs on the Nrf2 pathway were confirmed by RT-qPCR, and the results showed that SiO_2_ NPs increased the mRNA expressions of Nqo1, Nrf2, and HO-1 starting at week seven (Additional file 1 Figure S3a-d). The effects of Fumed FPs, Stober NPs, and Stober FPs on ROS production were also tested in this study. After 18 weeks of exposure, Stober NPs did not affect the mRNA expression of SOD1, SOD2, GSS, GCLC, or GCLM, but altered the levels of SOD, GSH, and MDA in sera and livers of mice (Fig. S3a-e). However, neither Fumed FPs nor Stober FPs affected the mRNA levels of T-SOD, GSH, or MDA in mice (Additional file 1 Figure S3e-i).

### SiO_2_ NPs induced ER stress in mice from week six

**Fig. 7:**
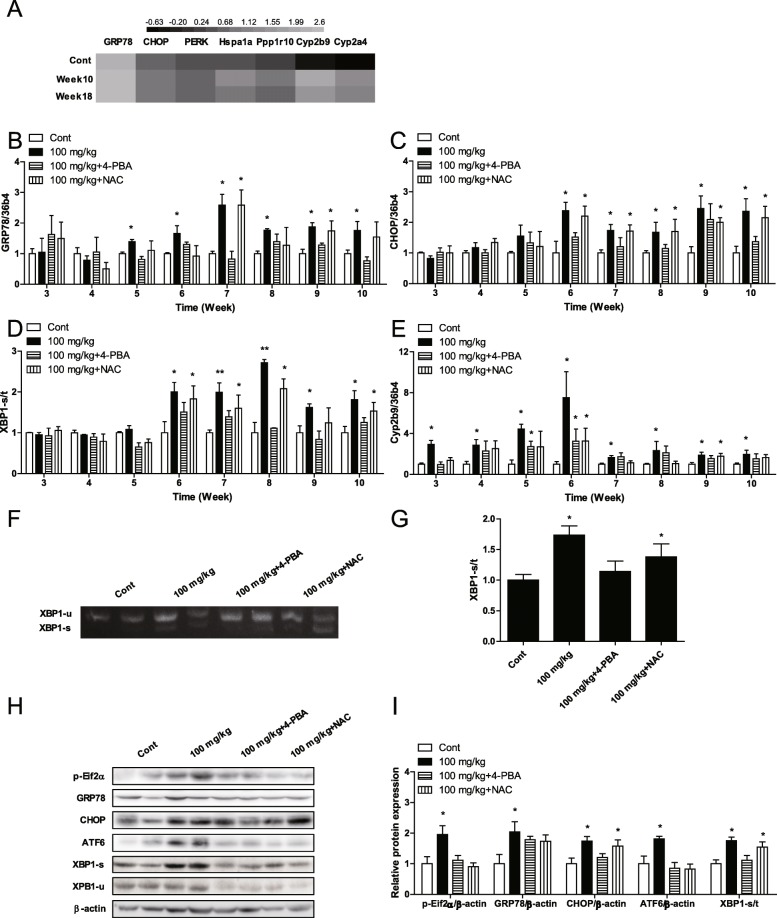
Oral administration of 100 mg/kg of SiO_2_ NPs (nanoparticles) induced ER stress starting at week six, and inhibition of ROS production did not reduce ER stress. **a** Fold change of genes associated with ER stress, as determined by RNA-seq. **b** Messenger RNA expression of GRP78. **c** Messenger RNA expression of CHOP. **d** The ratio of mRNA expression of sheared-XBP1/total-XBP1 (XBP1-s/t). **e** Messenger RNA expression of Cyp2b9. **f** AGE results of XBP1. **g** The ratio of XBP1-s/t in AGE results. **h** Protein expression of ER stress markers. **i** Ratios of ER stress markers. * *P* < 0.05, ** *P* < 0.01 vs. the control group. Results are the mean ± SE (*n* = 10)

RNA-seq results showed that SiO_2_ NPs affected genes related to ER stress in mice (Fig. 7a). RT-qPCR results showed that SiO_2_ NPs increased the mRNA expression of GRP78 and CHOP in the livers of mice starting at week six (Fig. 7b, c), and SiO_2_ NPs increased the ratio of sheared-XBP1/total-XBP1 (XBP1-s/t) in the livers of mice starting at week six (Fig. 7d). Cyp2b9 is a cytochrome P450 (CYP) enzyme involved in ER stress. In this study, SiO_2_ NPs increased the mRNA expression of Cyp2b9 in the livers of mice starting at week three (Fig. 7e). Furthermore, agarose gel electrophoresis results showed that SiO_2_ NPs increased the ratio of XBP1-s/t in the livers of mice at week six (Fig. 7f, g). Western blot results showed that SiO_2_ NPs increased the protein expression of phosphylated-eif2α, GRP78, CHOP, and ATF6, and increased the ratio of XBP1-s/t in the livers of mice at week six (Fig. 7h, i). These results showed that SiO_2_ NPs increased the expression of CYPs, then induced ER stress in mice starting at week six. In this study, 4-PBA significantly inhibited SiO_2_ NP-induced ER stress in mice. However, NAC did not affect SiO_2_ NP-induced ER stress in mice (Fig. 7b-i). After 18 weeks of exposure, Stober NPs increased the mRNA expression of GRP78, CHOP, XBP1-s/t, and Cyp2b9, and the protein expression of GRP78, CHOP, ATF6, and XBP1-s/t (Additional file 1Fig. S4a-c). However, Fumed FPs and Stober FPs did not induce the same changes.

### SiO_2_ NPs activated the NF-κB and MAPK pathways in mice

**Fig. 8:**
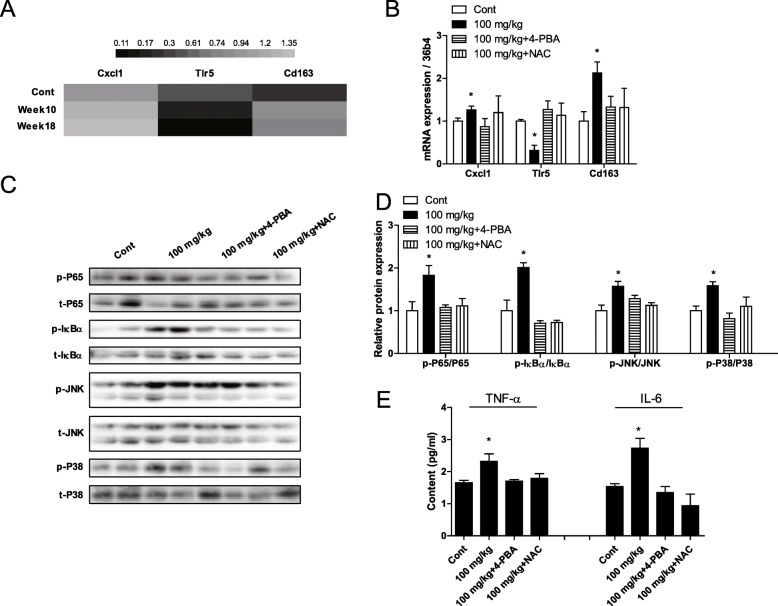
Relief of ER stress and reduction in ROS levels reduced activation of the NF-κB and MAPK pathways, and blunted the inflammatory response in the livers of mice following oral administration of 100 mg/kg bw of SiO_2_ NPs. **a** Fold change of genes related to inflammatory responses, as determined by RNA-seq. **b** Genes were validated by RT-qPCR. **c** Protein phosphorylation of NF-κB-p65, IκBα, JNK, and p38-MAPK. **d** Ratios of p-P65/P65, p-IκBα/ IκBα, p-JNK/JNK, and p-P38/P38. **e** Levels of TNF-α and IL-6 in mouse sera. * *P* < 0.05 vs. the control group. Results are the mean ± SE (*n* = 10)

RNA-seq results showed that SiO_2_ NPs affected genes related to inflammation (Fig. 8a). Real time qPCR data confirmed this result (Fig. 8b). Western blot results showed that SiO_2_ NPs activated the NF-κB pathway, through phosphorylation of p65-NF-κB and IκB (Fig. 8c, d). In addition, SiO_2_ NPs activated the MAPK pathway, which can result in IR, through phosphorylation of JNK and p38-MAPK (Fig. 8c, d). Furthermore, ELISA results showed that SiO_2_ NPs increased the levels TNF-α and IL-6 in sera of mice (Fig. 8e). In addition to 4-PBA- and NAC-mediated inhibition of ER stress and ROS generation, 4-PBA and NAC also inhibited SiO_2_ NP-induced inflammation and SiO_2_ NP-induced activation of the NF-κB and MAPK pathways (Fig. 8a-e). Furthermore, after 18 weeks of exposure, Stober NPs also activated the NF-κB and MAPK pathways, and also increased the levels of TNF-α and IL-6. In contrast, neither Fumed FPs nor Stober FPs induced these effects (Fig. S5a-d).

## Discussion

SiO_2_ NPs have been used for many years in food applications, such as for clearing of beers and wines, as anticaking agents to maintain flow properties in powder products, and to thicken pastes [1, 2]. The daily human intake of SiO_2_ NPs may be as high as 124 mg [3, 4]. In most in vivo toxicity studies, SiO_2_ NPs exhibit higher rates of absorption and more extensive organ distribution when administered orally than when delivered via other routes of administration [7, 9]. In addition, the acute toxicity and subacute toxicity after single or repeated doses via ingestion have been investigated, showing that SiO_2_ NPs affected nearly all organ systems [8, 9]. However, the toxicological effects of SiO_2_ NPs on blood glucose levels have not been studied. In this study, SiO_2_ NPs were administered orally to mice, and blood glucose was measured. Reports have shown that SiO_2_ NPs can be absorbed into the blood from the digestive system and accumulate in the liver, kidney, and spleen [7, 8, 19]. In the present study, oral administration of 25, 50, 100, and 200 mg/kg bw of fumed SiO_2_ NPs resulted in absorption and accumulation in the liver, pancreas, spleen, kidney, and small intestine of mice. Furthermore, fumed SiO_2_ NPs significantly increased blood glucose in mice at a dose of 100 mg/kg bw of SiO_2_ NPs or higher starting at week 10. Brun showed that the average intestinal surface area in humans is approximately 250 m^2^, and the average intestinal surface area of mice is 2.5 m^2^ [20]. Therefore, the average intestinal surface area of humans is 100-fold greater than that in mice. Human intake of 124 mg of SiO_2_ NPs (i.e., 0.5 mg/m^2^ intestine) corresponds to a 41.3 mg daily intake of a 0.03 kg mouse (1.25 mg/mouse, i.e., 0.5 mg/m^2^ intestine). A dose of 100 mg/kg bw (3 mg/mouse, i.e. 1.2 mg/m^2^ intestine), which increased blood glucose in mice in this study, corresponds to 2.5-fold the daily intake of humans. Whether a lifelong exposure of humans to SiO_2_ NPs can induce increased blood glucose should be evaluated further. Lifelong exposure of humans to SiO_2_ NPs likely results in accumulation of these nanoparticles, which is likely to lead to increased blood glucose. In this study, Stober SiO_2_ NPs were also absorbed from the digestive system, leading to increased blood glucose in mice starting at week 10. However, fumed SiO_2_ FPs and Stober SiO_2_ FPs were not absorbed from the digestive system and did not affect blood glucose levels in mice. Therefore, the main factor in SiO_2_ NPs effects on blood glucose was particle size. The World Health Organization uses the OGTT and ITT for diagnosis of diabetes types [21]. In this study, the OGTTs and ITTs were conducted in weeks 10 and 18, respectively. The results showed that the primary mechanism of SiO_2_ NP-induced hyperglycemia in both the early and late stages was IR. Hyperglycemia is the main characteristic of both diabetes subtypes [10, 22]. Type 1 diabetes is an autoimmune disease characterized by destruction of pancreatic β-cells, resulting in absolute insulin deficiency [14]. Insulin resistance, a feature of type 2 diabetes, is characterized by reduced activity of insulin despite increased insulin concentrations [22]. Insulin resistance leads to increased blood glucose, and high levels of blood glucose then induce the loss of β-cell mass, resulting in loss of function [23, 24]. In this study, SiO_2_ NPs did not induce apoptosis of β-cells, and thus did not affect insulin secretion. However, the mice exhibited reduced insulin sensitivity at weeks 10 and 18. Compared with SiO_2_ NPs, SiO_2_ FPs did not affect insulin secretion and did not induce IR in mice. The liver is the classical insulin-responsive organ, and is closely associated with IR [22]. In this study we evaluated the liver transcriptome to explore the mechanisms of SiO_2_ NP toxicity. Our results showed that SiO_2_ NPs affected several GO terms associated with ROS generation (GO-ID: 0055114, 16,491, 16,705, 4497, etc.) and ER stress (GO-ID: 34976, 6986, 34,620, 30,968, etc.). Reactive oxygen species production is a hallmark of oxidative damage in many diseases, and can lead to mitochondrial dysfunction, cellular aging, and apoptosis [13]. Reactive oxygen species also contribute to diabetes through induction of apoptosis in the pancreas, and induction of IR in liver. Silicon dioxide NPs have been reported to produce excessive ROS, resulting in consumption of endogenous antioxidants such as SOD and GSH, and damage to biological macromolecules such as nucleic acids, lipids, and proteins through lipid peroxidation [6, 8].. However, the mechanisms by which SiO_2_ NPs increase ROS levels in animals remains unclear. In this study, whole-genome network results showed that ROS generation was closely associated with ER stress. The ER is the cellular organelle responsible for synthesis and folding of secreted and membrane-bound proteins. A variety of stimuli can lead to accumulation of unfolded proteins, a condition called ER stress [14, 15]. PERK, IRE1, and ATF6 are important sensory elements and major injury pathway initiation factors of ER stress. When ER stress occurs, these proteins are separated from GRP78 to activate downstream signaling pathways, such as phosphorylation of eIF2α, mRNA and protein expression of CHOP, sheared-XBP1 (resulting in increased mRNA and protein ratios of sheared-XBP1/total XBP1 (XBP1-s/t)) and ATF6. Restoration of ER homeostasis occurs by reducing protein translation and promoting chaperone production [14, 16]. In addition, the resulting intermediates of ER stress can either activate or deactivate the expression of vital genes associated with ROS generation, such as those in the mitochondrial respiratory chain, the arachidonic acid pathway, the cytochrome P450 (CYP) family, glucose oxidase, amino acid oxidases, xanthine oxidase, NADPH/NADPH oxidases, and NO synthases [16, 17]. Furthermore, ROS can also cause ER stress. For example, CYP-derived peroxynitrite can react with H_2_O_2_ to generate singlet oxygen in the ER, resulting in ER stress [17, 25]. In this study, fumed SiO_2_ NPs induced ER stress (from week 6), which was followed by increased ROS (from week 8), and increased blood glucose in mice (from week 10). These results indicated that ROS did not induce ER stress in this study. Whole-genome results showed that SiO_2_ NPs increased the expression of Cyp2b9 and Cyp2a4. Cytochrome P450 enzymes, specifically CYP families 1–3, play a pivotal role in drug and xenobiotic metabolism [17, 25]. Overexpression of CYP enzymes in yeast and HepGH2 cells has been shown to induce responses involved in the URP and ER stress [16, 26]. In this study, SiO_2_ NPs increased the gene expression of Cyp2b9 earlier than fumed SiO_2_ NPs induced ER stress, which indicated that fumed SiO_2_ NPs activated xenobiotic metabolism, resulting in ER stress. Endoplasmic reticulum stress and ROS generation were each blocked by specific inhibitors. 4-phenylbutyric acid is a low molecular weight compound that stabilizes protein conformation, improves the folding capacity of the ER, and facilitates trafficking of mutant proteins to suppress ER stress [27]. In this study, 4-PBA inhibited fumed SiO_2_ NP-induced ER stress and inhibited ROS generation in mice, which suggested that fumed SiO_2_ NPs increased ROS production through ER stress. The transcriptional factor nuclear factor E2-related factor 2 (Nrf2), localized downstream of the PERK pathway, mediates antioxidant enzymes, resulting in augmentation of intracellular ROS production [15, 27]. In this study, whole-genome results showed that fumed SiO_2_ NP-induced ER stress affected the Nrf2 pathway by affecting the expression of Nrf2, Nqo-1, and HO-1, resulting in increased ROS production in mice. Stober NPs also affected the expression of Cyp2b9, ER stress, and ROS generation, but Fumed FPs and Stober FPs did not induce these effects. N-acetyl cysteine is a ROS scavenger [28]. However, NAC inhibited SiO_2_ NP-induced ROS production, but not ER stress. However, NAC inhibited increased blood glucose. These results showed that fumed SiO_2_ NPs induced ER stress, ER stress increased ROS production, and ROS induced blood glucose elevation. The major pathway of insulin receptor signal transduction is the insulin receptor substrate (IRS)-phosphatidylinositol 3-kinase (PI3K)-Akt (also known as PKB) pathway [29]. IRS1 isoforms are intracellular adaptor proteins that are recruited to the activated insulin receptor. Dephosphorylation of these isoforms on serine/threonine residues by the insulin receptor initiates the recruitment and activation of PI3K. Activated PI3K phosphorylates serine/threonine moieties of Akt. Akt acts as a central node in regulation of the biological effects of insulin [22, 29]. In this study, fumed SiO_2_ NP-induced ROS production inhibited dephosphorylation of the serine residues of IRS1 and inhibited phosphorylation of the serine residues of Akt, resulting in IR. In addition, fumed SiO_2_ NP-induced ROS production resulted in activation of the NF-κB and MAPK pathways, resulting in serine phosphorylation of IRS1. NF-κB is a family of transcription factors that plays a critical role in inflammation. Reactive oxygen species have been reported to induce NF-κB activation due to phosphorylation of IκB, which results in NF-κB translocation to the nucleus and phosphorylation of p65 [18, 27]. NF-κB induces the expression of inflammatory cytokines such as TNF-α and IL-6, which can result in phosphorylation of IRS1 [30, 31], resulting in activation of different mitogen-activated protein kinase (MAPK) cascades, leading to activation of p38 MAPK, extracellular regulated kinase (ERK), and c-Jun N-terminal kinase (JNK) [18, 29]. JNK and p38-MAPK also contribute to phosphorylation of IRS1 [22, 29]. Similar to Fumed NPs, Stober NPs also activated the NF-κB and MAPK pathways, resulting in IRS1 phosphorylation, and induction of IR in mice. In contrast, Fumed FPs and Stober FPs did not induce these effects.

## Conclusions

**Fig. 9:**
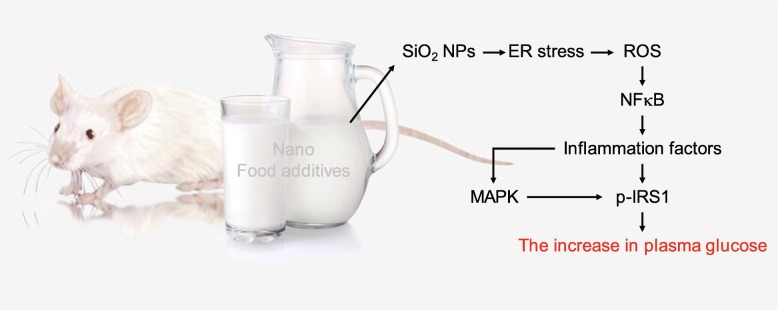
The nanofood additive SiO_2_ NPs entered the blood and accumulated in the liver and pancreas via the digestive tract of mice, resulting in activation of xenobiotic metabolism, ER stress, increased ROS production, and IR and increased blood glucose in mice

SiO_2_ NPs have been widely used as food additives. In this study, SiO_2_ NPs induced IR through ER stress and generation of ROS, but SiO_2_ FPs did not (Fig. 9). Administration of 100 mg/kg bw induced ER stress and increased blood glucose in this study, and corresponded to twice the estimated daily intake of humans. Lifelong exposure to SiO_2_ NPs may induce ER stress and increase blood glucose in humans. Therefore, this study strongly recommends the use of non-nanoformed SiO_2_ in food additives.

## Methods

### Preparation and characterization of SiO_2_ NPs

Fumed SiO_2_ NPs and FPs were obtained from Sigma Co., Ltd. (Product No. S5130, S5505, Roedermark, Germany). Stober SiO_2_ NPs were obtained from Zhongkeleiming Technology Co., Ltd. (Product No. DKSIO050, DKSIO300, Beijing, China). The primary particle sizes and morphology were measured using TEM and SEM (FEI Co., Ltd., OR, USA). The hydrodynamic size and zeta potential of nanoparticles in PBS were measured using DLS (Zetasizer, Malvern Instruments Ltd., Worcestershire, UK). Endotoxin analysis was performed using the Limulus Amoebocyte Lysate (LAL) assay and the Pyros Kinetix instrument from Associates of Cape Co., Ltd. (East Falmouth, MA).

### Animal care and exposure to SiO_2_ NPs

Six-week-old ICR male mice (22.97 ± 0.33 g) were obtained from Harbin Veterinary Research Institute (Harbin, China) and acclimated for 7 days after arrival at the study facility. Mice were housed in an animal room with controlled temperature (21–24 °C) and light cycle (12 h light/dark). Autoclaved water and rodent diet (Keao Co., Ltd., Beijing, China) were provided ad libitum. An ultrasonic bath (Ningbo Scientz Biotechnology Co., Ltd., Ningbo, China) at a frequency of 40 kHz with power at 400 W was used to scatter the SiO_2_ NPs and FPs. After ultrasonic wave stirring for 30 min, the SiO_2_ NP-PBS or SiO_2_ FP-PBS suspension was orally administered by syringe once per day, 7 days per week, for 10 or 18 weeks to each group of mice. To explore the endocrine effects of the oral administration of SiO_2_ NPs and determine the dose that increased blood glucose, mice were orally administered suspensions of SiO_2_ NPs at the doses of 25, 50, 100, and 200 mg/kg bw. The total doses the mice received were approximately 44.5, 96.7, 177.8, and 389.6 mg after 10 weeks, or approximately 86.7, 187.4, 355.3, 750.9 mg after 18 weeks. Then, in the experiment that explored the mechanism for the effect of SiO_2_ NPs on blood glucose, the dose of 100 mg/kg bw SiO_2_ NPs was selected. In addition, in the mechanism experiment, the doses of 100 mg/kg bw 4-PBA and NAC were orally administered to mice simultaneously with the oral administration of the suspension at 100 mg/kg bw SiO_2_ NPs. The total dose of mice received was approximately 179.7 mg after 10 weeks. The control group mice were given an equal volume of PBS.

### Blood collection and analysis

Tail vein blood was collected every 2 weeks. Before the collection, mice were fasted for 16 h. The blood glucose in the tail vein blood was measured using a glucose assay kit (Wako Pure Chemical Industries, Ltd., Osaka, Japan), and the blood insulin was measured using a mouse insulin ELISA kit (Shibayagi Co., Ltd., Gunma, Japan). TNF-α and IL-6 were measured using TNF-α and IL-6 kits, respectively (R&D Systems, Minneapolis, MN, USA).

### Glucose and insulin tolerance test

In OGTT, mice were fasted for 16 h and then orally administered glucose (1.5 g/kg bw). In ITT, mice were fasted for 6 h and then injected with insulin (0.4 IU/kg bw). Blood was collected for blood glucose and insulin measurement from the tail vein at 0, 30, 60, 120 min. Blood glucose and insulin were measured using each kit as above described.

### Silicon content analysis

The livers and pancreases were removed from the mice, and 0.3 g of each tissue was weighed, digested, and analyzed for silicon content. Briefly, the tissues were digested in nitric acid (ultrapure grade) overnight. After the addition of 0.5 mL of H_2_O_2_, the mixed solutions were heated at approximately 160 °C using a high-pressure reaction container in an oven chamber until the samples were completely digested. Then, the solutions were heated at 120 °C to remove the remaining nitric acid and until the solutions were colorless and clear. Last, the remaining solutions were diluted to 3 mL with 2% nitric acid. ICP-OES (Optima 5300 DV, Perkin Elmer Inc., CA, USA) was used to analyze the silicon concentration in the samples. The data are expressed as micrograms per gram fresh tissue. To assess whether SiO_2_ particles occurred in each tissue after the oral administration of SiO_2_ NPs, 0.1 g of each tissue was also weighed for SEM and EDXA. The tissues were homogenized in RIPA lysis buffer, filtered to discard cell debris using a 0.45 μm filter membrane, and centrifuged for 30 min at 18000×g to precipitate the SiO_2_ NPs. The precipitate was resuspended in alcohol and then spread out on an aluminum sheet, sputter-coated with platinum, and observed by SEM. The surface element was analyzed by EDXA.

### Reactive oxygen species assessment

ROS levels were assessed using the levels of total SOD (T-SOD), GSH and MDA. Livers were homogenized in nine volumes (1:10 w/v) of PBS. Homogenates were centrifuged at 750 g for 10 min at 4 °C to discard cell debris. The T-SOD, GSH and MDA of serum and liver supernatant were measured using each kit (Nanjing Jiancheng Bioengineering Institute, Nanjing, China).

### Immunofluorescence

Pancreases and livers were fixed in 4% paraformaldehyde solution for 24 h, embedded in paraffin, cut into 5 μm sections, and placed onto glass slides. The β-cell mass was identified by immunohistochemical localization of insulin with an anti-insulin antibody (Santa Cruz Biotechnology, Inc., CA, USA). For the analysis of cell apoptosis, pancreatic and liver sections were stained with the TUNEL (KeyGen Biotech. Co., Ltd., Nanjing, China). DNase I pretreated cells were used as the positive control.

### RNA sequencing and data analysis

To ensure unbiased analysis of tissue response, the total RNA was isolated from random sections (10–15 mg) of liver. The RNA was isolated using TRIzol reagent (Invitrogen, Carlsbad, CA, USA) and purified using a RNeasy Plus Mini kit (Qiagen, Mississauga, ON, Canada). Three livers of each group were sequenced, and the data were analyzed. In brief, the RNA was sequenced on one sequencing lane of an Illumina Genome Analyzer II system (Illumina). The paired-end reads were mapped to the mouse genome (version NCBIm37/mm9) using Tophat. The mapped reads were used to quantify the transcripts from the RefSeq reference database. The genes met the fold change > 2 and Benjamin Hochberg adjust Pvalue (B H Pvalue) < 0.05 were the significant differently expressed genes which were used for the following analysis. For the functional annotation analysis of genes, the BiNGO (http://apps.cytoscape.org/apps/bingo) was used. The GO terms met the B H *P*value < 0.001 were the significant GO terms.

### Real-time quantitative PCR

1 μg total RNA was used to perform reverse transcription using PrimeScript™ RT reagent Kit (TaKaRa, Tokyo, Japan). Real-time polymerase chain reaction amplification of cDNA was performed with SYBR Premix Ex Taq™ II (TaKaRa, Tokyo, Japan). Primers were listed in Table S3.

### Western blot

Liver tissues were resuspended in RIPA lysis buffer. Lysates were centrifuged for 15 min at 12000 g and 4 °C, and protein contents of the supernatant were determined using DC protein kit (Bio-Rad Laboratories, CA, USA). Aliquots of the proteins were separated by sodium dodecyl sulfate-polyacrylamide gel electrophoresis, and transferred to PVDF membranes (Bio-Rad Laboratories, CA, USA). PVDF membranes were incubated with antibodies against phospho-Eif2α, GRP78, CHOP, ATF6 (90KD), ATF6 (50KD), shear-XBP1 (XBP1-s), unshear-XBP1 (XBP1-u), Cleaved-Capsase3 (Cleaved-Casp3), phospho-IRS1 (Ser307), phospho-Akt (Ser473), IRS1, Akt, phospho-NFκB-p65, phospho-IκBα, phospho- JNK, phospho-p38-MAPK, NFκB-p65, IκBα, JNK1, p38-MAPK and β-actin. All antibodies were purchased from Cell Signaling Technology (Danvers, MA, USA).

### Statistical analysis

Results were expressed as mean ± SEM and were analyzed using GraphPad Prism version 5.0 (GraphPad Software, LaJolla, CA). All calculated significances are based on the one-way analysis of variance (ANOVA) test and the post hoc Tukey’s test. A *p* value less than 0.05 was considered statistically significant.

## Supplementary information

**Additional file 1: Figure S1-S5 (PDF 1327 kb)**

**Additional file 2: Table S1.** Significant differently expressed genes of RNA-seq (XLS 599 kb)

**Additional file 3: Table S2.** GO terms Table S1 (XLS 150 kb)

**Additional file 4: Table S3.** List of primer sequences (XLS 28 kb)

## Data Availability

The datasets used and/or analyzed during the current study are available from the corresponding author on reasonable request.

## Abbreviations

4-PBA: 4-phenylbutyric acid
ALP: alkaline phosphatase
AST: aspartate aminotransferase
B H Pvalue: Benjamin Hochberg adjust Pvalue
BP: Biological Process
bw: body weight
CC: Cellular Component
CYP: cytochrome P450
DLS: dynamic light scattering
EDXA: energy dispersive X-ray analysis
ER: endoplasmic reticulum
ERK: extracellular regulated kinase
GO: Gene Ontology
GSH: glutathione
HDL: high-density lipoprotein
ICP-MS: inductively coupled plasma-mass spectrometry
ICP-OES: inductively coupled plasma-optical emission spectrometry
IR: insulin resistance
IRS: insulin receptor substrate
JNK: c-Jun activating kinase
LDL: low-density lipoprotein
MAPK: mitogen-activated protein kinase
MDA: malonyl dialdehyde
MF: Molecular Function
NAC: N-acetylcysteine
NF-κB: Nuclear factor-κB
NPs: nanoparticles
Nrf2: nuclear factor E2-related factor 2
OGTT: Oral Glucose Tolerance Test
PI3K: phosphatidylinositol 3-kinase
ROS: reactive oxygen species
SEM: scanning electron microscopy
SiO_2_: Silicon dioxide nanoparticles SOD superoxide dismutase
TEM: transmission electron
TUNEL: terminal deoxynucleotidyl transferase-mediated dUDP nick end-labeling
XBP1-s/t: shear-XBP1/total XBP1
