# Supplementary material for: Silicon dioxide nanoparticles induce insulin resistance through endoplasmic reticulum stress and generation of reactive oxygen species
Source: Part Fibre Toxicol. 2019 Nov 7;16:41. doi: 10.1186/s12989-019-0327-z (PMC6836410; doi:10.1186/s12989-019-0327-z)
Supplement: Supplementary file 1 — Additional file 1: Figure S1-S5 (PDF 1327 kb) [file 12989_2019_327_MOESM1_ESM.pdf]

Figure S1

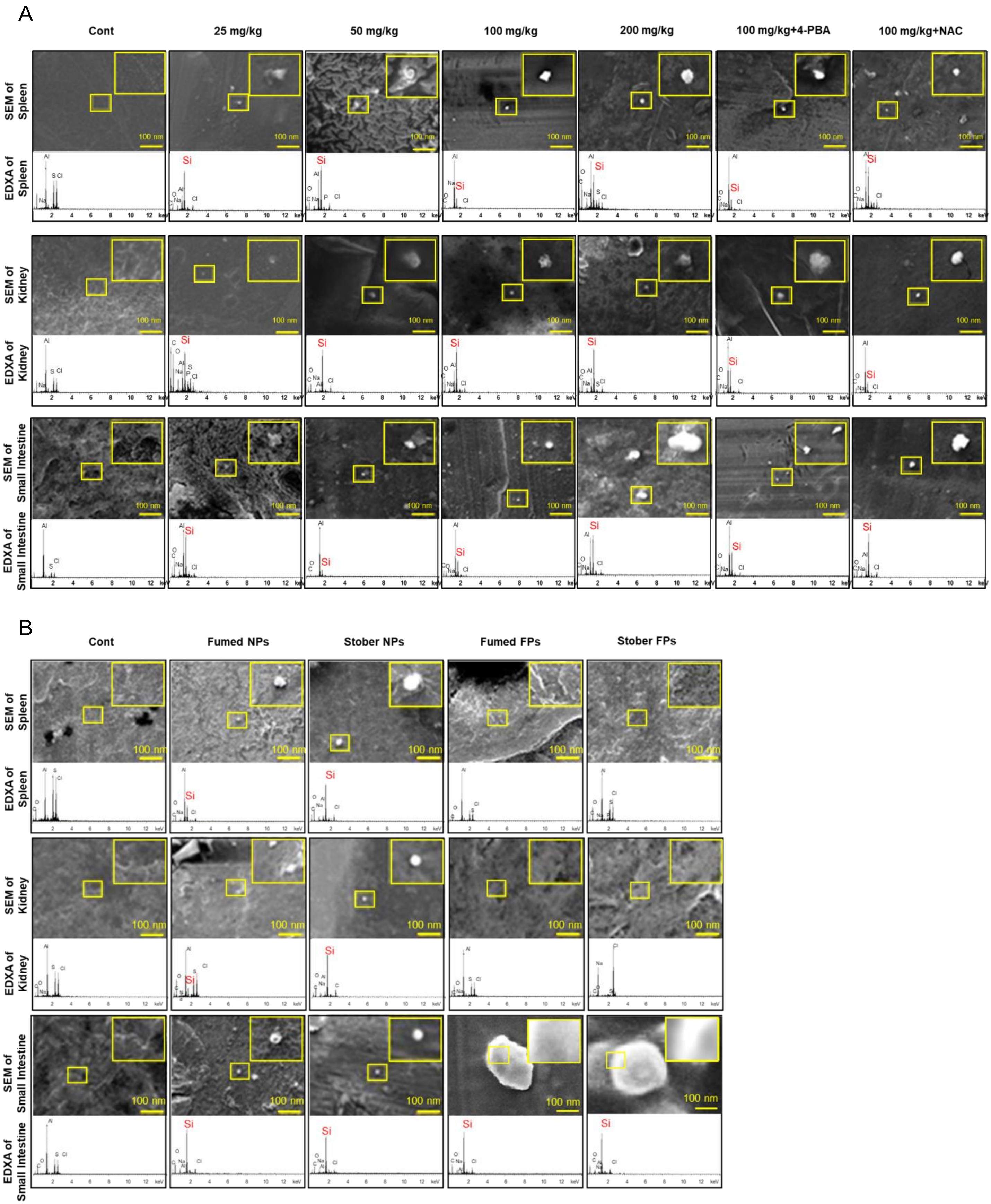

Figure S1. SEM (nanosized and finesized spherical white objects are SiO_2 NPs and FPs) and EDXA (arrows indicate silicon) of tissue homogenates. (A) SEM and EDXA of mice orally administrated with different dose of fumed SiO_2 NPs. (B) SEM and EDXA of mice orally administrated with fumed SiO_2 NPs, stober SiO_2 NPs, fumed SiO_2 FPs, stober SiO_2 FPs.

Figure S2

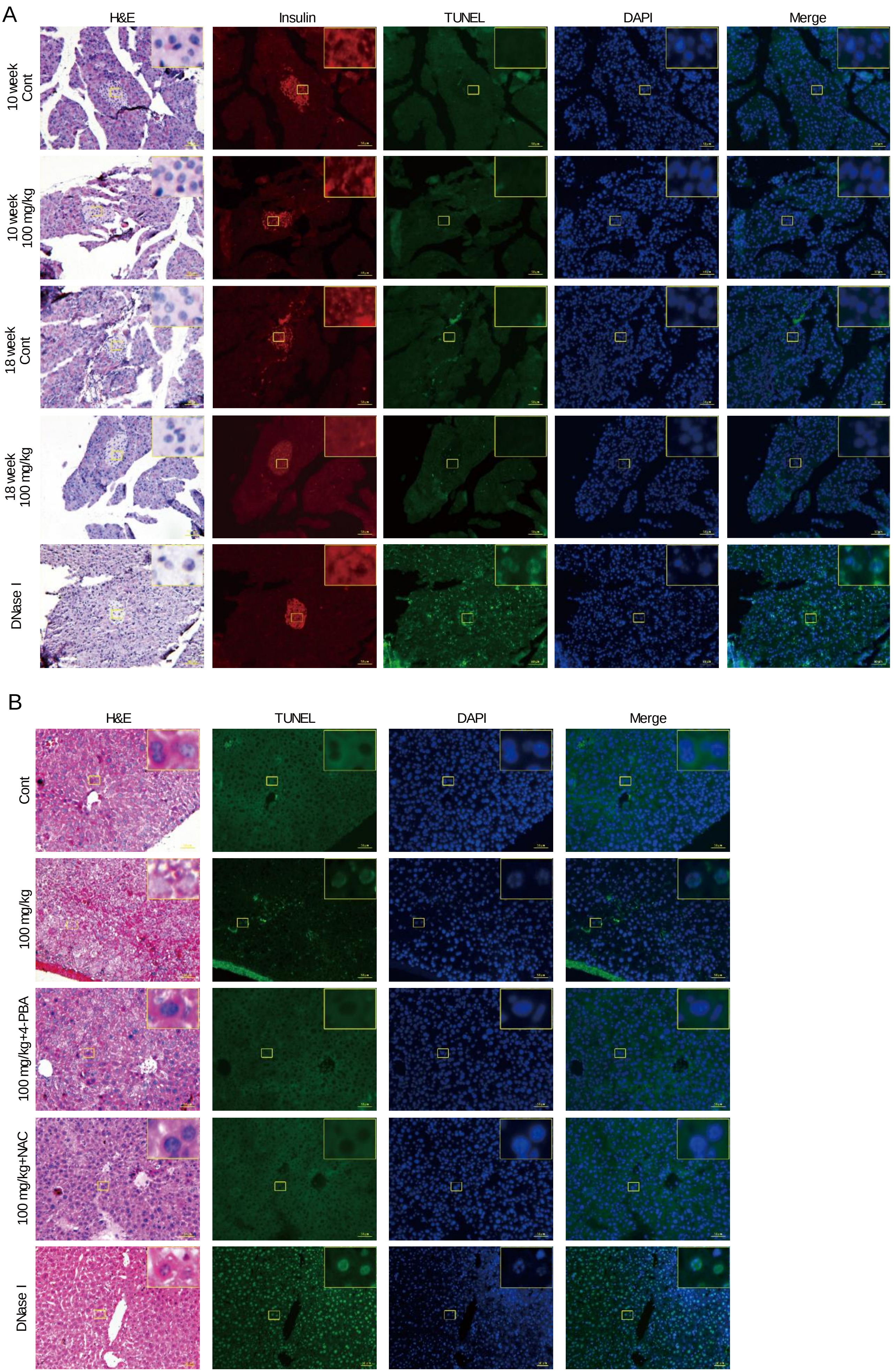
A
H&E
Insulin
TUNEL
DAPI
Merge
10 week Cont
10 week 100 mg/kg
18 week Cont
18 week 100 mg/kg
DNase I
B
H&E
TUNEL
DAPI
Merge
Cont
100 mg/kg
100 mg/kg+4-PBA
100 mg/kg+NAC
DNase I

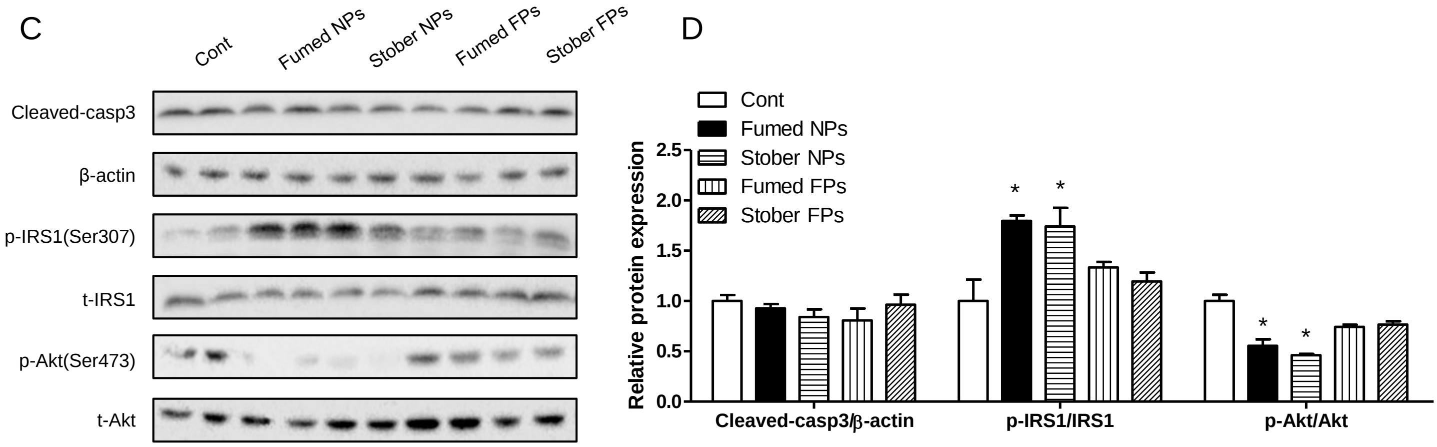

Figure S2. Effects of fumed SiO_2 NPs, stober SiO_2 NPs, fumed SiO_2 FPs and stober SiO_2 FPs on apoptosis and protein expression and protein phosphorylation. (A) Apoptosis of mouse pancreas cells (Insulin (red), TUNEL (green), and Nucleus (blue) co-staining). (B) Apoptosis of mouse liver cells (TUNEL (green) and nucleus (blue) co-staining). (C) Protein expression of Cleaved-caspase 3, and protein phosphorylation of IRS1 and Akt. (D) Ratios of Cleaved-caspase 3/β-actin, p-IRS1/IRS1 and p-Akt/Akt. * $P < 0.05$ vs. the control group. Results are the mean ± SE (n = 10).

Figure S3

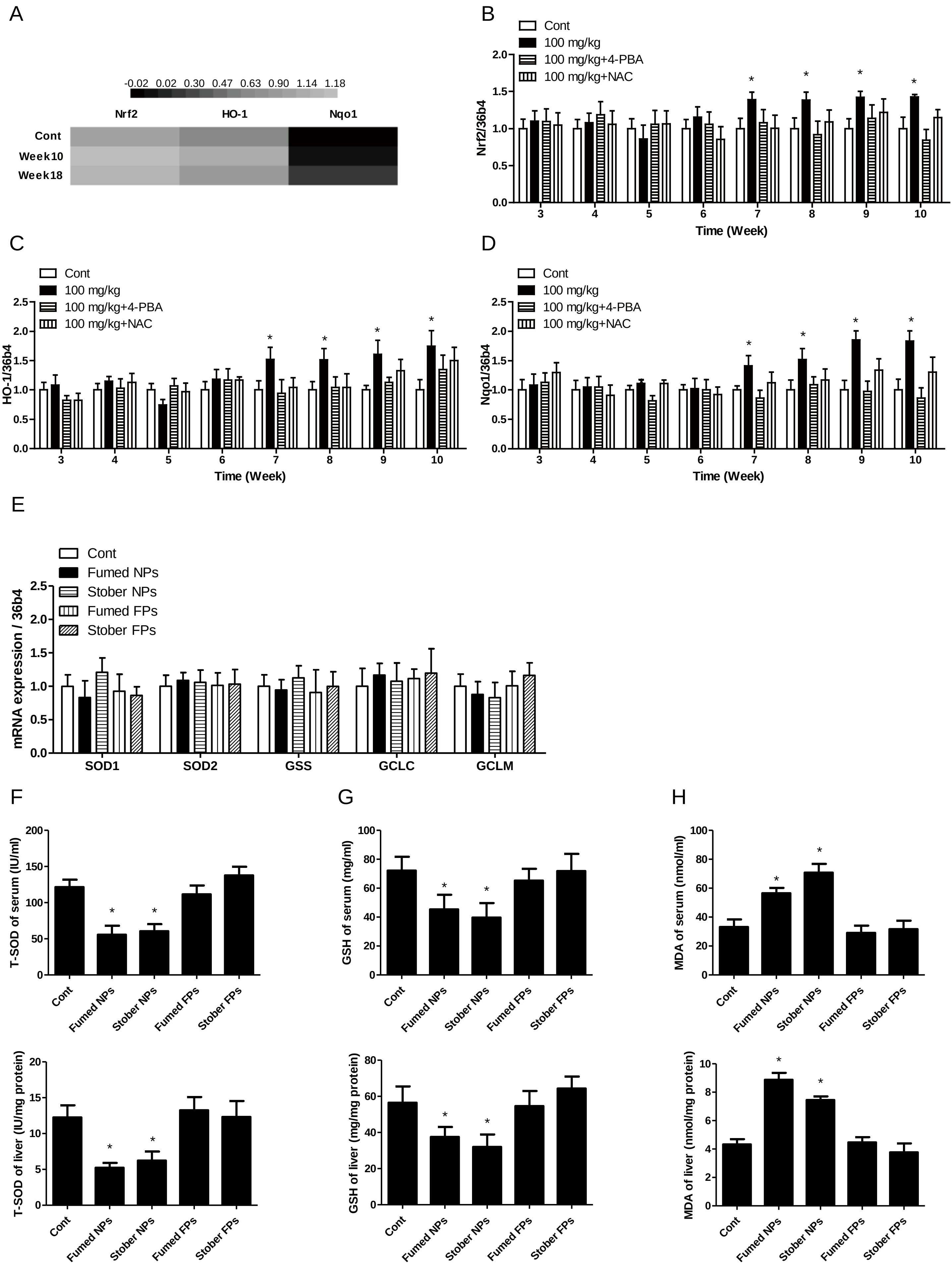

A
-0.02 0.02 0.30 0.47 0.63 0.90 1.14 1.18
Nrf2
HO-1
Nqo1
Cont
Week10
Week18
B
Cont
100 mg/kg
100 mg/kg+4-PBA
100 mg/kg+NAC
Nrf2/36b4
Time (Week)
C
HO-1/36b4
D
Nqo1/36b4
E
Cont
Fumed NPs
Stober NPs
Fumed FPs
Stober FPs
mRNA expression / 36b4
SOD1
SOD2
GSS
GCLC
GCLM
F
T-SOD of serum (IU/ml)
T-SOD of liver (IU/mg protein)
G
GSH of serum (mg/ml)
GSH of liver (mg/mg protein)
H
MDA of serum (nmol/ml)
MDA of liver (nmol/mg protein)

I

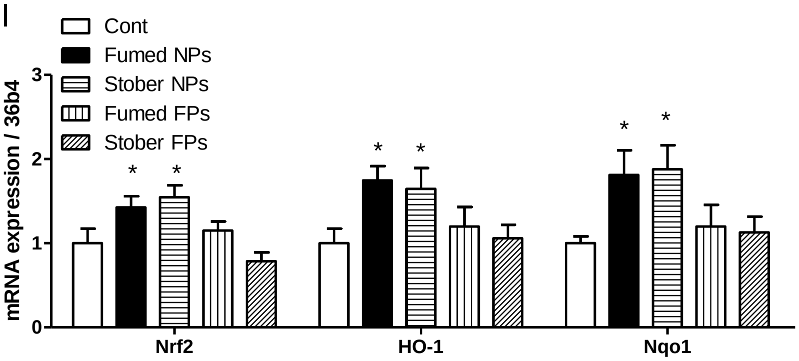

Figure S3. Oral administration of 100 mg/kg SiO_2 NPs increased plasma ROS levels, but oral administration of the same dose of SiO_2 FPs did not. (A) Fold changes of genes in the Nrf2 pathway, based on RNA-seq results. (B) Messenger RNA expression of Nrf2. (C) Messenger RNA expression of HO-1. (D) Messenger RNA expression of Nqo-1. (E) RT-qPCR results for ROS-related genes. (F) Levels of T-SOD in sera and livers. (G) Levels of GSH in sera and livers. (H) Levels of MDA in sera and livers. (I) RT-qPCR results for Nrf2 pathway genes. * $P < 0.05$ vs. the control group. Results are the mean ± SE (n = 10).

Figure S4

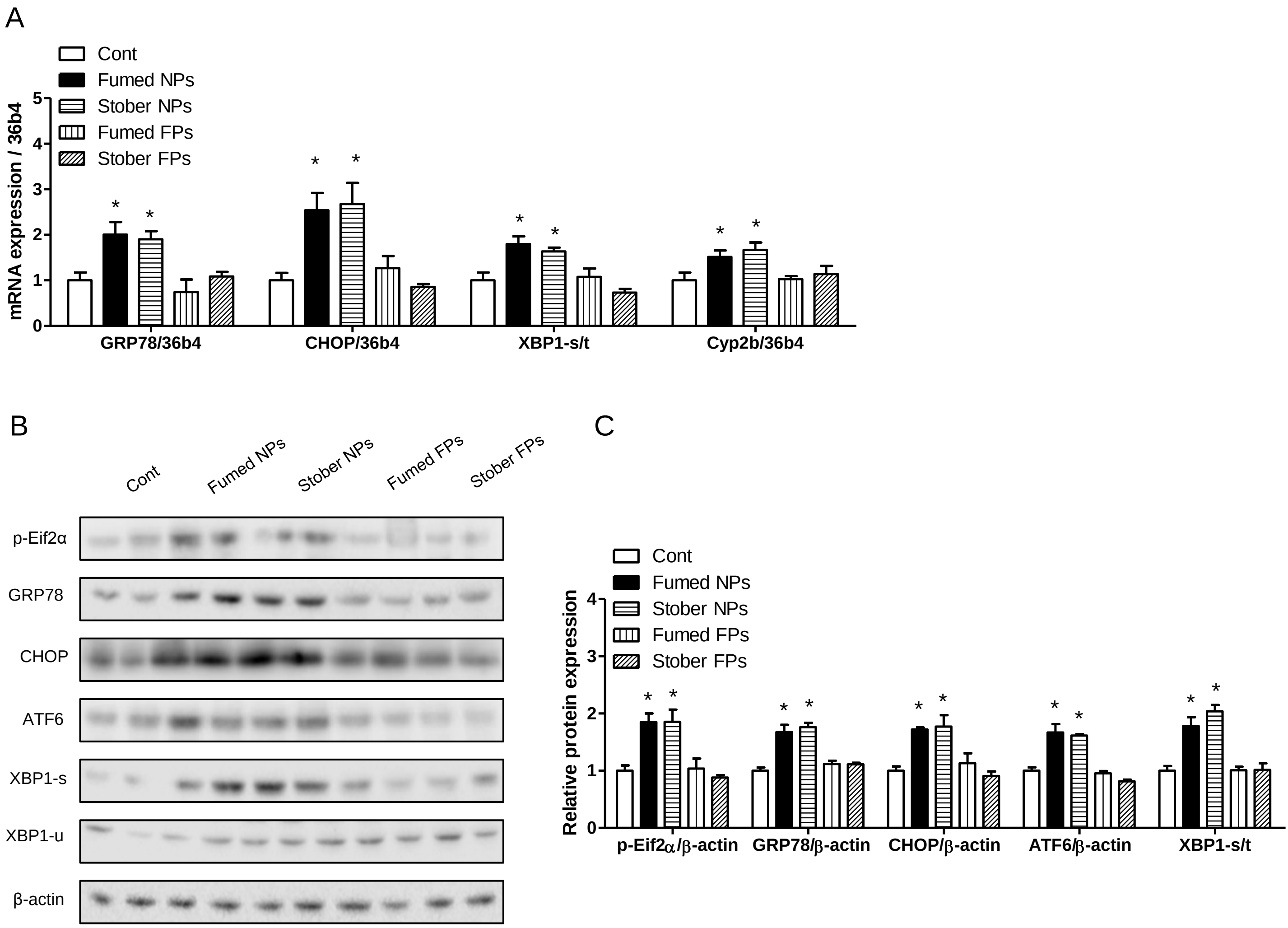

Figure S4. Oral administration of 100 mg/kg SiO_2 NPs induced ER stress, but oral administration of the same dose of SiO_2 FPs did not. (A) RT-qPCR results for ER stress-related genes. (B) Protein expression of ER stress markers. (C) Ratios of ER stress markers. * $P < 0.05$ vs. the control group. Results are the mean ± SE (n = 10).

Figure S5

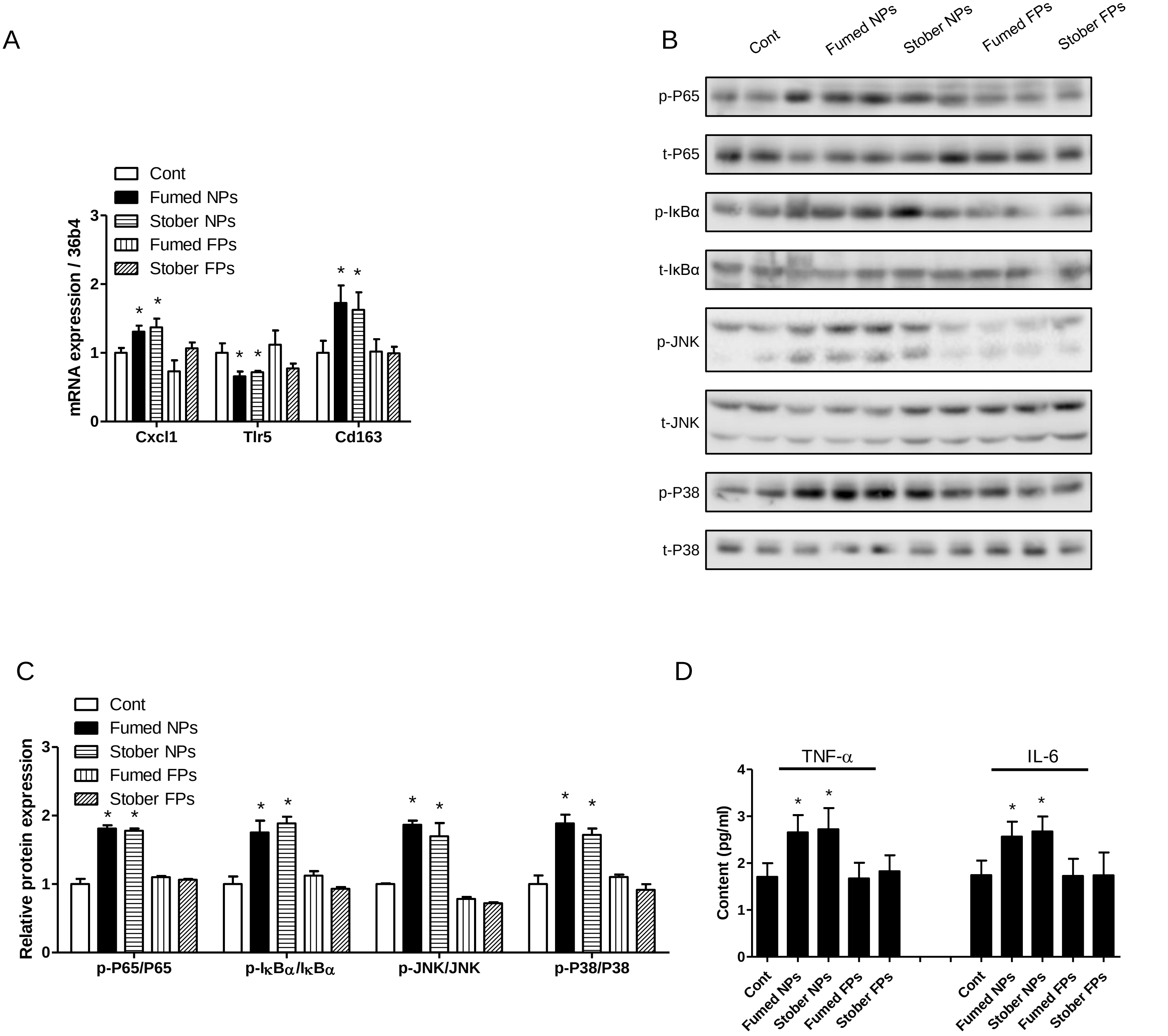

Figure S5. Oral administration of 100 mg/kg SiO_2 NPs activated the NF-κB and MAPK pathways and induced inflammation response in livers of mice, but oral administration of the same dose of SiO_2 FPs did not. (A) RT-qPCR results for inflammation response-related genes. (B) Protein phosphorylation of NF-κB-p65, IκBα, JNK, and p38-MAPK. (C) Ratios of p-P65/P65, p-IκBα/IκBα, p-JNK/JNK, and p-P38/P38. * $P < 0.05$ vs. the control group. Results are the mean ± SE (n = 10).
